# High prevalence of breastmilk‐acquired cytomegalovirus infection in jaundiced infants

**DOI:** 10.1002/jcla.23199

**Published:** 2020-01-29

**Authors:** Juanjuan Hou, Juan Liu, Yingfang Fan, Hongjun Zheng, Haiyan Zhao, Jianmin Yang, Jiamin Yan, Yi Ma, Xia Liu, Juan Li, Xiaoni Jia, Peisong Chen

**Affiliations:** ^1^ Department of Clinical Laboratory Medicine The People Hospital of Qingyang City Qingyang China; ^2^ Department of Infectious Disease The People Hospital of Qingyang City Qingyang China; ^3^ Department of Obstetrics The People Hospital of Qingyang City Qingyang China; ^4^ Department of Neonatology The People Hospital of Qingyang City Qingyang China; ^5^ Department of Clinical Laboratory Medicine The First Affiliated Hospital of Sun Yat‐sen University Sun Yat‐sen University Guangzhou China

**Keywords:** breastmilk, cytomegalovirus, jaundiced infants, urine

## Abstract

**Background:**

Our objective was to evaluate the prevalence and different diagnostic methods of breastmilk (BM)‐acquired cytomegalovirus (CMV) infection in a pathologically jaundiced cohort.

**Methods:**

A total of 400 infants confirmed with pathological jaundice at The People's Hospital of Qingyang City were screened for BM‐acquired CMV infection between February 2018 and February 2019. A total of 300 infants were finally enrolled in our study. CMV infection was confirmed by detecting both CMV‐DNA in various samples using FQ‐PCR and CMV‐IgM with chemiluminescence. Clinical and other laboratory data were collected from these infants during their hospitalization or regular visits.

**Results:**

Ninety‐eight (32.67%) subjects were confirmed to be BM CMV‐DNA–positive, and 18 (18.37%) were diagnosed with a BM‐acquired CMV infection. All 18 (100%) infants with a BM‐acquired CMV infection were CMV‐DNA–positive in urine, while 5 (27.78%) cases and 11 (61.11%) cases were confirmed in plasma and peripheral blood mononuclear cells (PBMCs), respectively. Only 6 (33.33%) infants were CMV‐IgM–positive. Birthweight, direct bilirubin, aspartate aminotransferase, and the viral load in BM of the BM‐acquired CMV group were higher than those in the non‐infected group (*P* < .05). Low birthweight and viral load in BM were risk factors for BM‐acquired CMV infection. Detecting CMV‐DNA in urine samples exhibited better performance than the other methods for screening BM‐acquired CMV infections.

**Conclusions:**

Our study found a high prevalence of BM‐acquired CMV infection in jaundiced infants, and detecting CMV‐DNA in a urine sample was the most sensitive method for disease screening.

## 1 INTRODUCTION

Cytomegalovirus (CMV) (formal name: human herpesvirus 5) infection is common in China, and a primary CMV infection often occurs in infants.^1^, ^2^ CMV has the biological properties of latency and activation, and the infection remains throughout life. As a weak pathogen, CMV is not usually pathogenic in the population with normal immune function, and most cases of CMV infection are asymptomatic. CMV generally causes serious consequences in the immunosuppressed population, particularly fetuses and neonates with developmental immunodeficiencies.^3^, ^4^, ^5^, ^6^ CMV infection is frequently seen in mothers, and CMV can be excreted via breastmilk (BM) in 13%‐27% of cases. BM is the best source of nutrition for neonates. The American Academy of Pediatrics has reported that BM feeding is very important for babies ≤6 months old, and BM is irreplaceable, particularly in premature infants; thus, CMV infections via BM are worthy of research attention.^7^, ^8^

As a common disease during the neonatal period, neonatal jaundice is mostly a physiological phenomenon during the normal growth process, but is occasionally a clinical manifestation of some latent disease. As shown by clinical analysis, pathological jaundice is mostly caused by an infection, of which the most common is a CMV infection.^9^, ^10^, ^11^ A BM‐acquired CMV infection usually causes no clinical symptoms in healthy neonates and has a low probability of causing deafness and nervous system sequela. However, the risk of serious sequela from CMV may increase in pathologically jaundiced infants.^12^, ^13^ BM‐acquired CMV infection in pathologically jaundiced infants is seldom reported, and there are no reported data concerning local populations. CMV screening and diagnostic methods should be comprehensively assessed to facilitate selecting a suitable test method.

## 2 MATERIALS AND METHODS

### 2.1 Study population

**Figure 1:**
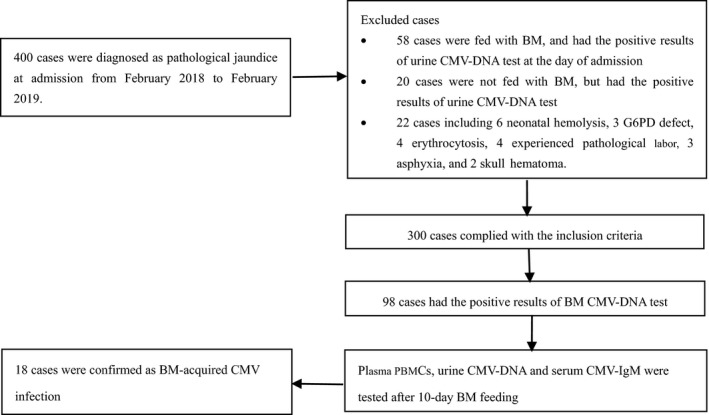
Flowchart defining the cohort (all 300 neonates were fed fresh BM in our study)

A total of 400 infants who were diagnosed with pathological jaundice in the Department of Neonatology of Qingyang People's Hospital from February 2018 to February 2019 were selected. The diagnostic criteria were as follows^14^: (a) jaundice <24 hours after birth; (b) total bilirubin (TBIL) >the 95th percentile on the hourly bilirubin curve; (c) TBIL increase >0.2 mg/dL/h (3.4 μmol/L/h); (d) full‐term neonates with jaundice 2 weeks after birth; and (e) TBIL < 5 mg/dL (86 μmol/L) and direct bilirubin (DBIL) >1 mg/dL (17 μmol/L), or TBIL > 5 mg/dL (86 μmol/L) and DBIL > 20% of TBIL. The exclusion criteria were as follows: (a) BM feeding and with positive urine CMV‐DNA test results on the day of admission; (b) no BM feeding, but with a positive urine CMV‐DNA test result (congenital CMV infection); and (c) diseases, such as neonatal hemolysis, a glucose‐6‐phosphate dehydrogenase (G6PD) defect, erythrocytosis, pathological labor, asphyxia, and skull hematoma, as indicated by relevant examinations after the admission (Figure 1). In this study, the neonates were all fed fresh BM.

### 2.2 Methods

#### 2.2.1 Diagnosis of CMV infection

The diagnosis of CMV infection can be performed in the following ways: (a) exclusion of congenital CMV infection—urine CMV‐DNA test was performed at admission, and there was no definite infection <2 weeks after birth; (b) BM CMV(+)—CMV‐DNA > 500 copies/mL in the BM fed to pathologically jaundiced infants was detected by real‐time fluorescence quantitative polymerase chain reaction( RT‐FQ‐PCR); (c) BM‐acquired CMV infection^3^, ^15^—after 2 weeks of CMV(+) BM feeding in non‐congenital CMV infection infants, CMV infection was indicated by positive plasma CMV‐DNA, peripheral blood mononuclear cell (PBMC) CMV‐DNA, urine CMV‐DNA, or serum CMV‐IgM; (d) CMV‐IgM test—performed with a fully automatic chemiluminescence analyzer and matching reagents (Liaison); (e) CMV‐DNA test—the reagents (Hunan Sansure Biotech Inc) and RT‐FQ‐PCR method (ABI 7500 fluorescence PCR amplifier; Applied Biosystems) were used; CMV‐DNA was extracted, amplified, and analyzed strictly according to the procedures stipulated in the kit manual; and (f) a positive result was defined as a typical S growth curve of CMV‐DNA, and the laboratory results showed CMV‐DNA > 500 copies/mL; otherwise, the result was negative. CMV‐IgM ≥ 18 U/mL indicated positive, and CMV‐IgM < 18 U/mL indicated negative. All laboratory data were obtained under conditions of a negative control, a positive control, and an indoor quality control.

#### 2.2.2 Processing of different types of samples before the CMV‐DNA test

##### Urine sample

10 mL of mixed urine from three urinations (including urina sanguinis) was collected from subjects, sealed, and sent for examination. After mixing, 1 mL of the urine sample was centrifuged at 12 000 rpm for 5 minutes, and the supernatant was discarded.

##### BM sample

3 mL of BM was collected into a sterile tube. After mixing, 1 mL BM was centrifuged at 1500 rpm for 10 minutes, and the white upper lipid layer was discarded; 500 μL of the intermediate layer was added to a 200 μL virus enrichment solution and centrifuged at 12 000 rpm for 5 minutes, and the supernatant was discarded.

##### PBMC sample

1 mL venous blood was collected into a EDTA‐anticoagulated tube and then proportionally diluted with 1 mL sterile normal saline. The diluted solution was slowly added to 1 mL of lymphocyte separating solution and then subjected to density gradient centrifugation at 1,500 rpm for 20 minutes. The intermediate layer of monocytes was collected into a centrifuge tube and centrifuged at 12 000 rpm for 5 minutes, and the supernatant was discarded.

##### Plasma samples

2 mL venous blood was collected into a EDTA‐anticoagulated tube, after centrifugation at 4000 rpm for 10 minutes, and 100 μL of plasma was added to 100 μL of the virus enrichment solution and centrifuged at 12 000 rpm for 5 minutes. The supernatant was discarded.

#### 2.2.3 Collection of blood samples and tests of the laboratory

##### The venous blood was drawn

TBIL, DBIL, adenosine deaminase (ADA), total bile acid (TBA), alkaline phosphatase (ALP), alanine aminotransferase (ALT), aspartate aminotransferase (AST), and gamma‐glutamyltransferase (γ‐GGT) were detected with an AU2700 full‐automatic biochemical analyzer (Japan); the complete cell count was measured with a Mindray BC‐5800 blood cell analyzer.

##### The reference limits for infants were as follows

TBIL 0‐27 μmol/L, DBIL 0‐8.55 μmol/L, ADA 4‐24 U/L, TBA 0.5‐10 μmol/L, ALP 45‐125 U/L, ALT 9‐50 U/L, AST 15‐40 U/L, γ‐GGT 10‐60 U/L, white blood cell (WBC) 15‐20 × 10^9^/L, and platelets (PLT) 100‐300 × 10^9^/L. When the test results exceeded the upper limit of the references, an increase in the indices was indicated.

### 2.3 Ethics statement

Written informed consent was signed by the parents of each infant. This study was approved by the Research Ethics Committee of the People's Hospital of Qingyang City and conformed to the 1975 Declaration of Helsinki and its later amendments.

### 2.4 Data analysis

Data were analyzed with SPSS (version 20.0) software (SPSS Inc). Proportions were calculated by the chi‐square test or Fisher's exact test, and numerical data were assessed by the *t* test. The diagnostic performance was assessed by the area under the receiver operating characteristic curve (AUC). In this study, an AUC > 0.7 was considered useful. Sensitivity, specificity, positive predictive value (PPV), and negative predictive value (NPV) were calculated. *P*‐values < .05 were considered significant.

## 3 RESULTS

### 3.1 Demographic data of the subjects

Finally, 300 pathologically jaundiced infants were enrolled, including 178 males and 122 females, with a male/female ratio of 1.46:1 and an age of 3‐21 days (mean: (15.00 ± 8.00) days). Among all subjects, 98 cases (32.67%) were BM CMV(+), including eight cases of low‐birthweight infants (8.16%), 13 cases of hepatosplenomegaly (13.27%), four cases of neonatal septicemia (4.08%), eight cases of anemia (8.16%), 15 cases of neonatal pneumonia (15.31%), five cases of hypoxic‐ischemic encephalopathy (5.10%), three cases of bronchopulmonary dysplasia (3.06%), three cases of congenital heart disease (3.06%), and other cases of no obvious abnormality. Among the 98 BM CMV (+) pathologically jaundiced infants, 18 cases (18.37%) were confirmed to be BM‐acquired CMV infections.

### 3.2 Positive rate of BM‐acquired CMV infection in different types of samples

In the 18 infants with BM‐acquired CMV infection, there were 18 cases of urine CMV‐DNA (+) (100%), five cases of plasma CMV‐DNA (+) (27.78%), and 11 cases of PBMCs CMV‐DNA (+) (61.11%). All cases of plasma CMV‐DNA (+) were PBMC CMV‐DNA (+). Six (33.33%) serum CMV‐IgM (+) infants demonstrated urine CMV‐DNA (+) and PBMC CMV‐DNA (+).

### 3.3 Comparison of clinical characteristics and laboratory indices between the BM‐acquired CMV‐infected and non‐infected groups

The birthweight, DBIL, AST, and viral load in BM were significantly different between the BM‐acquired CMV‐infected and non‐infected groups (*P* < .05). However, age, gestational age, onset time of jaundice, TBIL, ADA, TBA, ALP, ALT, γ‐GGT, WBC, and PLT were not significantly different between these groups (*P* > .05; Table 1).

**Table 1:** Comparison of clinical characteristics and laboratory indices between the BM‐acquired CMV‐infected and non‐infected groups (mean ± SD)

	CMV + VE (n = 18)	CMV ‐VE(n = 80)	*P*‐value
Age (d)	11.53 ± 2.34	10.21 ± 1.98	.945
Gestational age (wk)	38.90 ± 7.20	39.40 ± 10.90	.798
Birthweight (kgs)	2. 30 ± 0.98	3.54 ± 1.17	.047*
Onset of jaundice (d)	5.01 ± 1.16	5.93 ± 1.78	.075
TBil (μmol/L)	351.87 ± 47.45	302.24 ± 31.11	.068
DBil (μmol/L)	90.45 ± 23.87	36.29 ± 10.40	.037*
ADA (U/L)	13.26 ± 2.72	11.46 ± 3.93	.945
TBA (μmol/L)	40.34 ± 16.57	43.26 ± 10.15	.089
ALP (U/L)	367.44 ± 43.12	315.32 ± 67.45	.067
ALT (U/L)	62.69 ± 18.46	69.01 ± 15.34	.891
AST (U/L)	296.35 ± 34.98	178.66 ± 23.55	.044*
γ‐GGT (U/L)	397.24 ± 67.23	477.6 0 ± 56.01	.062
WBC (10^9^/L)	18.82 ± 3.11	17.27 ± 4.02	.889
PLT (10^9^/L)	189.23 ± 20.17	231.26 ± 36.24	.059
Viral load in BM (log copies/mL)	5.98 ± 1.13	3.90 ± 0.89	.040*

* *P*‐values with statistical significance are highlighted in the BM‐acquired CMV‐infected groups (<0.05 significant).

### 3.4 Risk factors for BM‐acquired CMV infection

The following possible risk factors for BM‐acquired CMV infection were analyzed: small‐for‐gestational‐age infants, low‐birthweight infants, premature rupture of fetal membranes, BM feeding start age, onset time of jaundice, blood transfusion history, and BM CMV viral load. The probability of a low‐birthweight infant and the BM CMV viral load were significantly higher in the infected group than those in the non‐infected group (*P* < .05). No significant differences were observed in small‐for‐gestational‐age infants, premature rupture of the fetal membranes, BM feeding start age, onset time of jaundice, and blood transfusion history between the two groups (*P* > .05).

### 3.5 Correlations between the BM CMV‐DNA (+) rate and the plasma CMV‐DNA (+), PBMC CMV‐DNA (+), urine CMV‐DNA (+), and serum CMV‐IgM (+) rates

The laboratory results of 98 BM CMV‐DNA (+) samples were as follows: plasma CMV‐DNA (−) (93 cases), PBMC CMV‐DNA (−) (87 cases), urine CMV‐DNA (−) (80 cases), and serum CMV‐IgM (−) (92 cases). The 202 BM CMV‐DNA (−) samples were as follows: plasma CMV‐DNA (+) (0 cases), PBMC CMV‐DNA (+) (one case), urine CMV‐DNA (+) (two cases), and serum CMV‐IgM (+) (one case). As results, the BM CMV‐DNA (+) rate was correlated with the plasma CMV‐DNA (+), PBMC CMV‐DNA (+), urine CMV‐DNA (+), and serum CMV‐IgM (+) rates (*r* = 0.187, *P* = .001; *r* = 0.257, *P* < .001; *r* = 0.329, *P* < .001; *r* = 0.175, *P* < .001; Table 2).

**Table 2:** Correlations between the BM CMV‐DNA (+) rate and the plasma CMV‐DNA (+), PBMCs CMV‐DNA (+), urine CMV‐DNA (+), and serum CMV‐IgM (+) rates (n = 300)

BM CMV‐DNA	Plasma CMV‐DNA		PBMCs CMV‐DNA		Urine CMV‐DNA		Serum CMV‐IgM	
	Positive	Negative	Positive	Negative	Positive	Negative	Positive	Negative
Positive (n = 98)	5	93	11	87	18	80	6	92
Negative (n = 202)	0	202	1	201	2	200	1	201
	5	295	12	288	20	280	7	293

### 3.6 Diagnostic performance of the different test methods for BM‐acquired CMV infection

As shown in Table 3, the diagnostic performance for BM‐acquired CMV infection was achieved best by the urine CMV‐DNA test with the maximum AUC, better by the PBMCs CMV‐DNA test. The diagnostic value of plasma CMV‐DNA and serum CMV‐IgM was similar, which showed high specificity but lack of sensitivity.

**Table 3:** Diagnostic performance of the different test methods for BM‐acquired CMV infection

	Sensitivity	Specificity	PPV	NPV	AUC (95% CI)
Plasma CMV‐DNA	27.78	100.00	100.00	86.02	0.639 (0.536‐0.734)
PBMCs CMV‐DNA	61.11	98.75	91.67	91.80	0.799 (0.706‐0.873)
Urine CMV‐DNA	100.00	97.50	90.00	100.00	0.988 (0.941‐0.999)
Serum CMV‐IgM	33.33	98.75	85.71	86.81	0.660 (0.558‐0.753)

## 4 DISCUSSION

About 30%‐50% of neonatal CMV infections are caused by recurrent infection in pregnant women, and the pathogenesis of BM‐acquired CMV infection may be relevant to immunodeficiency of the infants. The BM‐acquired CMV infection rates in premature and low‐birthweight infants differ among countries; however, there are few data about BM‐acquired CMV infection in jaundiced neonates.^16^, ^17^, ^18^ Our study results show that the BM CMV‐DNA (+) rate was 32.67% (98/300 cases) and the BM‐acquired CMV infection rate was 18.37% (18/98 cases) among the 300 pathologically jaundiced infants. There were particular proportions of low‐birthweight infants, hepatosplenomegaly, neonatal septicemia, anemia, neonatal pneumonia, hypoxic‐ischemic encephalopathy, bronchopulmonary dysplasia, and congenital heart disease in the BM CMV (+) infants, indicating that immunity of the BM CMV (+) infants was lower than that of the BM CMV (−) infants; meanwhile, the CMV infection rate in China is relatively higher, so accompanied by an increase in the BM‐acquired CMV infection rate.^1^, ^2^ Some active substances are produced in breastfeeding mothers, and they accumulate in the mammary glandular cells to promote CMV replication; therefore, the CMV infection rate is higher in infants fed with virus‐bearing BM, which suggests that BM feeding is an important pathway for postnatal mother‐infant CMV transmission.^19^, ^20^ In addition, neonatal jaundice is mostly caused by CMV; thus, jaundiced neonates should receive the routine CMV test.

This study demonstrated that birthweight, DBIL, AST, and viral load in BM increased significantly in the infected group, and the differences between the infected group and the non‐infected group were significant (*P* < .05). A single‐factor analysis showed a higher risk for BM‐acquired CMV infection in jaundiced infants with a lower birthweight and a higher BM CMV viral load, which is basically the same as the results of Martins‐Celini.^21^ Gunkel et al revealed that the severity of CMV infection is associated with extremely preterm babies (gestational age <26 weeks).^22^ Josephson et al reported that BM is the main source for neonatal CMV infection after birth. The viral load in the BM of CMV‐transmitting mothers is obviously higher than that of non–CMV‐transmitting mothers.^19^ These findings indicate that closer clinical attention should be paid to BM‐acquired CMV infections. Early prevention, close monitoring of infection, early diagnosis, and active treatment are necessary to achieve a good prognosis in pathologically jaundiced infants with high‐risk factors.

No strong evidence has been provided in clinical studies considering the efficacy, safety, and adverse reactions of anti‐HCMV drugs for treating neonates^3^. In this study, among 18 pathologically jaundiced infants with BM‐acquired CMV infections, 11 cases had mild clinical symptoms except for a high jaundice index and they were not treated with antiviral drugs. The infants' condition was relieved after symptomatic treatment, such as regular intermittent blue light exposure combined with an ursodeoxycholic acid tablet (10 mg/kg/d). Bilirubin returned to normal from the follow‐up to 8 months after discharge. The other 7 children with severe clinical symptoms (3 with hearing impairment, 2 with severe pneumonia, 1 with septicemia, and 1 with congenital heart disease) were referred to the superior children's hospital for treatment, so they were lost to their follow‐up visits.

Accurate test methods are very important for diagnosing a CMV infection. In this study, samples of five types including BM, plasma, PBMCs, urine, and serum were tested, and the results showed that the CMV‐positive rate in BM and urine samples was significantly higher than that of the PBMCs, plasma, and serum samples. The BM CMV‐DNA (+) rate was correlated with plasma CMV‐DNA (+), PBMC CMV‐DNA (+), urine CMV‐DNA (+), and serum CMV‐IgM (+) rates, indicating that the BM CMV‐DNA test is necessary for infants with negative blood or urine test results and a suspected CMV infection, which can guide the tracing and treatment of CMV infection‐associated diseases.^23^ We also evaluated the diagnostic performance of the four methods and determined that the best diagnostic performance for BM‐acquired CMV infection was achieved by the urine CMV‐DNA test, better by the PBMC CMV‐DNA test. The mixed urine of three urinations (including urina sanguinis) was used as the urine sample, so the highest CMV detection rate in the urine was observed in our study, which may be attributed to the extremely high invasion of CMV into renal epithelial cells and the detection of a high‐concentration CMV‐DNA in urine when CMV is at the active replicating stage in infants. According to Gunkel et al, a urine sample is superior to a salivary sample for diagnosing postnatal CMV infection in premature infants.^24^ Exler et al showed that positive salivary samples and especially weakly positive CMV‐DNA must be confirmed by the urine CMV‐DNA test; the urine CMV‐DNA test is the gold standard for diagnosing congenital CMV infection in infants.^25^ Despite that saliva samples test is an easy and important method for the screening of CMV infection, further confirmation with CMV DNA test in urine or blood samples is necessary.^26^ Our study results are consistent with those of previous studies. Blood collection is inconvenient and painful for neonates, while urine and BM samples are collected more easily; therefore, urine and BM CMV‐DNA tests are recommended for primary screening of neonates with a CMV infection. Moreover, CMV intermittently releases toxins, and CMV samples not in the toxin‐releasing state will not conform to the clinical diagnosis; therefore, repeated, continuous, and multiple tests are suggested to improve the detection rate of CMV infection.^4^, ^27^

As shown by many studies, freezing the BM does not eliminate the risk of CMV infection, and the immune activity of BM changes after pasteurization; thus, fresh BM is still encouraged for feeding.^28^, ^29^, ^30^ In this study, all pathologically jaundiced infants were fed fresh BM. We should inform parents of the corresponding risks to infants with BM‐acquired CMV infection and suggest that long‐term examinations, such as CBC, a liver function examination, a physical examination, and hearing and nerve functional development tests, be given to infants. This study had some limitations that may have influenced the results: (a) The sample size was small, and the period of our study was not long enough to enroll more subjects. (b) All subjects were local permanent residents who lived in areas of relatively poor economic and sanitary conditions. (c) The lower limit of our RT‐FQ‐PCR was only 500 copies/mL. Currently, there are more sensitive methods for the detection of CMV‐DNA but were not adopted due to an economic limitation.

## 5 CONCLUSION

A high prevalence of BM‐acquired CMV infection was detected in jaundiced infants. Detecting CMV‐DNA in a urine sample was the most sensitive method for screening the disease.
